# 5-Fluoro­isophthalic acid

**DOI:** 10.1107/S1600536811004004

**Published:** 2011-02-09

**Authors:** Jin-Ling Mi, Le Chen, Ming-Yang He

**Affiliations:** a Key Laboratory of Fine Petrochemical Technology, Changzhou University, Changzhou 213164, People’s Republic of China

## Abstract

In the crystal structure of the title compound, C_8_H_5_FO_4_, the complete molecule is generated by crystallographic twofold symmetry with two C atoms and the F atom lying on the axis. The mol­ecule is almost planar with the carboxyl group twisted with respect to the mean plane of the benzene ring by a dihedral angle of 2.01 (1)°. In the crystal, inter­molecular O—H⋯O hydrogen bonds and C—H⋯F inter­actions connect the mol­ecules into a two-dimensional supra­molecular array.

## Related literature

For isophthalic acid, see: Bhogala *et al.* (2005 ▶); Derissen (1974 ▶). For the use of the title compound in crystal engin­eering, see: Zhang *et al.* (2010 ▶).
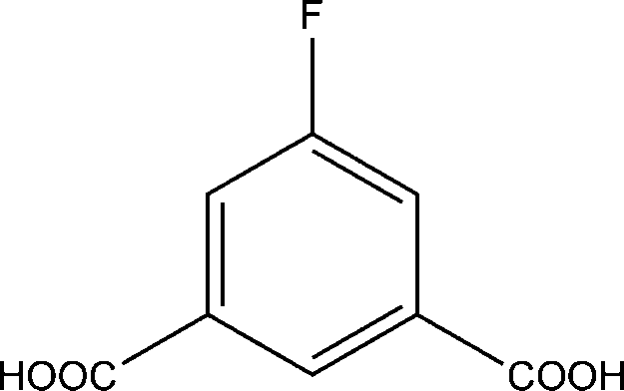


## Experimental

### 

#### Crystal data

C_8_H_5_FO_4_
*M* *_r_* = 184.12
Monoclinic, 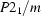

*a* = 3.7736 (8) Å
*b* = 16.292 (4) Å
*c* = 6.2753 (14) Å
β = 91.871 (5)°
*V* = 385.60 (14) Å^3^
*Z* = 2
Mo *K*α radiation
μ = 0.14 mm^−1^
*T* = 297 K
0.22 × 0.20 × 0.15 mm

#### Data collection

Bruker APEXII CCD diffractometer
Absorption correction: multi-scan (*SADABS*; Sheldrick, 2003 ▶) *T*_min_ = 0.969, *T*_max_ = 0.979
2201 measured reflections
743 independent reflections
603 reflections with *I* > 2σ(*I*)
*R*_int_ = 0.018

#### Refinement

*R*[*F* ^2^ > 2σ(*F* ^2^)] = 0.041
*wR*(*F* ^2^) = 0.161
*S* = 1.04
743 reflections
64 parameters
H-atom parameters constrained
Δρ_max_ = 0.16 e Å^−3^
Δρ_min_ = −0.15 e Å^−3^

### 

Data collection: *APEX2* (Bruker, 2003 ▶); cell refinement: *SAINT* (Bruker, 2001 ▶); data reduction: *SAINT*; program(s) used to solve structure: *SHELXS97* (Sheldrick, 2008 ▶); program(s) used to refine structure: *SHELXL97* (Sheldrick, 2008 ▶); molecular graphics: *SHELXTL* (Sheldrick, 2008 ▶) and *DIAMOND* (Brandenburg, 2005 ▶); software used to prepare material for publication: *SHELXTL*.

## Supplementary Material

Crystal structure: contains datablocks I, global. DOI: 10.1107/S1600536811004004/go2002sup1.cif

Structure factors: contains datablocks I. DOI: 10.1107/S1600536811004004/go2002Isup2.hkl

Additional supplementary materials:  crystallographic information; 3D view; checkCIF report

## Figures and Tables

**Table 1:** Hydrogen-bond geometry (Å, °)

*D*—H⋯*A*	*D*—H	H⋯*A*	*D*⋯*A*	*D*—H⋯*A*
O1—H1⋯O2^i^	0.82	1.81	2.625 (2)	174
C5—H5⋯F1^ii^	0.93	2.52	3.404 (2)	160

Symmetry codes: (i) 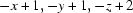; (ii) 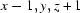.

**Fig. 1.**
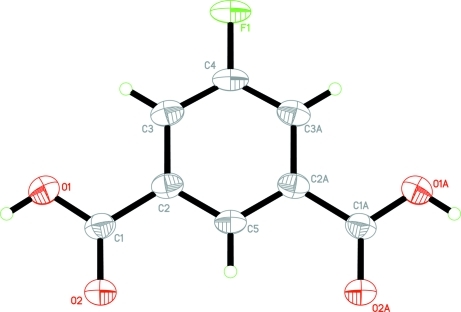
The molecular structure of the title compound drawn with 30% probability ellipsoids.

**Fig. 2.**
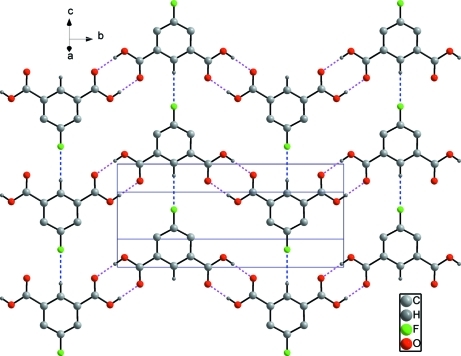
Two-dimensional hydrogen-bonded layer of the title compound. Hydrogen bonds are indicated as dashed lines.

## supplementary crystallographic information

### Comment

As an analogue of isophthalic acid (Bhogala *et al.* 2005; Derissen, 1974), 5-fluoroisophthalic acid has been seldom used in the crystal engineering of organic or inorganic-organic systems (Zhang *et al.* 2010). The fluorinated group may participate in hydrogen-bonding and may also induce luminescence properties. Herein we report the crystal structure of the title compound, C_8_H_5_FO_4_, to further investigate the supramolecular interactions involving the fluorine atom. The structure of the title compound, is shown below. The molecule presents *C*_2 _symmetry with the fundamental unit lying on a *C*_2_-axis at [*x*, 3/4, *z*]. Intermolecular O—H···O interactions between adjoining centrosymmetry-related carboxylic groups form a hydrogen-bonded ribbon running along the [010] direction. C—H···F interactions connect the ribbons into a two-dimensional supramolecular array.

### Experimental

5-Fluoroisophthalic acid and solvents for synthesis and analysis were commercially available and used as received. Single crystals suitable for X-ray diffraction were obtained by slow evaporation of the methanol solution of the title compound.

### Refinement

Benzene H atoms were assigned to calculated positions with C—H = 0.93 Å, and refined using a riding model, with *U*iso(H) = 1.2*U*eq(C). H atoms bound to carboxylic O atoms were located in difference maps and refined as riding with *U*_iso_(H) = 1.5 *U*_eq_(O).

### Crystal data

**Table:**

C_8_H_5_FO_4_	*F*(000) = 188
*M**_r_* = 184.12	*D*_x_ = 1.586 Mg m^−^^3^
Monoclinic, *P*2_1_/*m*	Mo *K*α radiation, λ = 0.71073 Å
Hall symbol: -P 2yb	Cell parameters from 1020 reflections
*a* = 3.7736 (8) Å	θ = 2.5–28.0°
*b* = 16.292 (4) Å	µ = 0.14 mm^−^^1^
*c* = 6.2753 (14) Å	*T* = 297 K
β = 91.871 (5)°	Block, colourless
*V* = 385.60 (14) Å^3^	0.22 × 0.20 × 0.15 mm
*Z* = 2	

### Data collection

**Table:**

Bruker APEXII CCD diffractometer	743 independent reflections
Radiation source: fine-focus sealed tube	603 reflections with *I* > 2σ(*I*)
graphite	*R*_int_ = 0.018
φ and ω scans	θ_max_ = 25.5°, θ_min_ = 2.5°
Absorption correction: multi-scan (*SADABS*; Sheldrick, 2003)	*h* = −4→4
*T*_min_ = 0.969, *T*_max_ = 0.979	*k* = −17→19
2201 measured reflections	*l* = −7→5

### Refinement

**Table:**

Refinement on *F*^2^	Primary atom site location: structure-invariant direct methods
Least-squares matrix: full	Secondary atom site location: difference Fourier map
*R*[*F*^2^ > 2σ(*F*^2^)] = 0.041	Hydrogen site location: inferred from neighbouring sites
*wR*(*F*^2^) = 0.161	H-atom parameters constrained
*S* = 1.04	*w* = 1/[σ^2^(*F*_o_^2^) + (0.1244*P*)^2^] where *P* = (*F*_o_^2^ + 2*F*_c_^2^)/3
743 reflections	(Δ/σ)_max_ < 0.001
64 parameters	Δρ_max_ = 0.16 e Å^−^^3^
0 restraints	Δρ_min_ = −0.15 e Å^−^^3^

### Special details

**Table:**

Geometry. All esds (except the esd in the dihedral angle between two l.s. planes) are estimated using the full covariance matrix. The cell esds are taken into account individually in the estimation of esds in distances, angles and torsion angles; correlations between esds in cell parameters are only used when they are defined by crystal symmetry. An approximate (isotropic) treatment of cell esds is used for estimating esds involving l.s. planes.
Refinement. Refinement of F^2^ against ALL reflections. The weighted R-factor wR and goodness of fit S are based on F^2^, conventional R-factors R are based on F, with F set to zero for negative F^2^. The threshold expression of F^2^ > 2sigma(F^2^) is used only for calculating R-factors(gt) etc. and is not relevant to the choice of reflections for refinement. R-factors based on F^2^ are statistically about twice as large as those based on F, and R- factors based on ALL data will be even larger.

### Fractional atomic coordinates and isotropic or equivalent isotropic displacement parameters (Å^2^)

**Table:**

	*x*	*y*	*z*	*U*_iso_*/*U*_eq_	
C1	0.6268 (4)	0.59855 (9)	0.8588 (3)	0.0524 (5)	
C2	0.7105 (4)	0.67633 (9)	0.7484 (2)	0.0487 (5)	
C3	0.8602 (4)	0.67563 (10)	0.5486 (3)	0.0529 (5)	
H3	0.9107	0.6265	0.4806	0.063*	
C4	0.9312 (5)	0.7500	0.4549 (3)	0.0540 (6)	
C5	0.6370 (5)	0.7500	0.8476 (3)	0.0477 (6)	
H5	0.5379	0.7500	0.9813	0.057*	
F1	1.0787 (4)	0.7500	0.2629 (2)	0.0744 (6)	
O1	0.7073 (4)	0.53205 (8)	0.7622 (2)	0.0775 (6)	
H1	0.6348	0.4905	0.8205	0.116*	
O2	0.4837 (4)	0.60037 (7)	1.0328 (2)	0.0722 (6)	

### Atomic displacement parameters (Å^2^)

**Table:**

	*U*^11^	*U*^22^	*U*^33^	*U*^12^	*U*^13^	*U*^23^
C1	0.0490 (9)	0.0612 (10)	0.0474 (10)	−0.0023 (6)	0.0088 (7)	−0.0080 (7)
C2	0.0385 (8)	0.0652 (11)	0.0424 (9)	−0.0012 (6)	0.0022 (6)	−0.0051 (6)
C3	0.0420 (9)	0.0726 (12)	0.0441 (10)	−0.0006 (6)	0.0027 (7)	−0.0087 (7)
C4	0.0426 (11)	0.0852 (16)	0.0346 (11)	0.000	0.0069 (9)	0.000
C5	0.0400 (10)	0.0642 (14)	0.0395 (11)	0.000	0.0072 (8)	0.000
F1	0.0745 (10)	0.1105 (12)	0.0393 (8)	0.000	0.0190 (7)	0.000
O1	0.1022 (11)	0.0610 (8)	0.0715 (10)	−0.0050 (6)	0.0359 (8)	−0.0121 (6)
O2	0.0938 (10)	0.0622 (9)	0.0628 (9)	−0.0038 (6)	0.0355 (7)	−0.0032 (5)

### Geometric parameters (Å, °)

**Table:**

C1—O2	1.235 (2)	C3—H3	0.9300
C1—O1	1.2826 (19)	C4—F1	1.343 (2)
C1—C2	1.483 (2)	C4—C3^i^	1.377 (2)
C2—C5	1.3841 (18)	C5—C2^i^	1.3841 (19)
C2—C3	1.392 (2)	C5—H5	0.9300
C3—C4	1.377 (2)	O1—H1	0.8201
			
O2—C1—O1	123.73 (15)	C2—C3—H3	121.1
O2—C1—C2	119.91 (13)	F1—C4—C3	118.37 (11)
O1—C1—C2	116.35 (15)	F1—C4—C3^i^	118.36 (11)
C5—C2—C3	120.34 (15)	C3—C4—C3^i^	123.3 (2)
C5—C2—C1	118.83 (15)	C2—C5—C2^i^	120.3 (2)
C3—C2—C1	120.83 (14)	C2—C5—H5	119.9
C4—C3—C2	117.89 (16)	C2^i^—C5—H5	119.9
C4—C3—H3	121.1	C1—O1—H1	113.5
			
O2—C1—C2—C5	2.3 (3)	C1—C2—C3—C4	179.97 (14)
O1—C1—C2—C5	−178.51 (16)	C2—C3—C4—F1	179.40 (14)
O2—C1—C2—C3	−177.68 (14)	C2—C3—C4—C3^i^	−0.3 (3)
O1—C1—C2—C3	1.5 (3)	C3—C2—C5—C2^i^	0.3 (3)
C5—C2—C3—C4	0.0 (3)	C1—C2—C5—C2^i^	−179.72 (12)

Symmetry codes: (i) *x*, −*y*+3/2, *z*.

### Hydrogen-bond geometry (Å, °)

**Table:**

*D*—H···*A*	*D*—H	H···*A*	*D*···*A*	*D*—H···*A*
O1—H1···O2^ii^	0.82	1.81	2.625 (2)	174
C5—H5···F1^iii^	0.93	2.52	3.404 (2)	160

Symmetry codes: (ii) −*x*+1, −*y*+1, −*z*+2; (iii) *x*−1, *y*, *z*+1.
